# Analysis of the potential relationship between COVID-19 and Behcet’s disease using transcriptome data

**DOI:** 10.1097/MD.0000000000033821

**Published:** 2023-05-17

**Authors:** Zhibai Zhao, Chenyu Zhou, Mengna Zhang, Ling Qian, Wenhui Xia, Yuan Fan

**Affiliations:** a Key Laboratory of Shaanxi Province for Craniofacial Precision Medicine Research, College of Stomatology, Xi’an Jiaotong University, Xi’an, China; b Department of General Dentistry, College of Stomatology, Xi’an Jiaotong University, Xi’an, China; c Department of Oral Mucosal Diseases, The Affiliated Stomatological Hospital of Nanjing Medical University, Nanjing, China; d Jiangsu Province Key Laboratory of Oral Diseases, Nanjing, China.

**Keywords:** Behcet’s disease, bioinformatics technology, COVID-19, peripheral blood mononuclear cells, SARS-CoV-2

## Abstract

To investigate the potential role of COVID-19 in relation to Behcet’s disease (BD) and to search for relevant biomarkers. We used a bioinformatics approach to download transcriptomic data from peripheral blood mononuclear cells (PBMCs) of COVID-19 patients and PBMCs of BD patients, screened the common differential genes between COVID-19 and BD, performed gene ontology (GO) and pathway analysis, and constructed the protein-protein interaction (PPI) network, screened the hub genes and performed co-expression analysis. In addition, we constructed the genes-transcription factors (TFs)-miRNAs network, the genes-diseases network and the genes-drugs network to gain insight into the interactions between the 2 diseases. We used the RNA-seq dataset from the GEO database (GSE152418, GSE198533). We used cross-analysis to obtain 461 up-regulated common differential genes and 509 down-regulated common differential genes, mapped the PPI network, and used Cytohubba to identify the 15 most strongly associated genes as hub genes (ACTB, BRCA1, RHOA, CCNB1, ASPM, CCNA2, TOP2A, PCNA, AURKA, KIF20A, MAD2L1, MCM4, BUB1, RFC4, and CENPE). We screened for statistically significant hub genes and found that ACTB was in low expression of both BD and COVID-19, and ASPM, CCNA2, CCNB1, and CENPE were in low expression of BD and high expression of COVID-19. GO analysis and pathway analysis was then performed to obtain common pathways and biological response processes, which suggested a common association between BD and COVID-19. The genes-TFs-miRNAs network, genes-diseases network and genes-drugs network also play important roles in the interaction between the 2 diseases. Interaction between COVID-19 and BD exists. ACTB, ASPM, CCNA2, CCNB1, and CENPE as potential biomarkers for 2 diseases.

## 1. Introduction

COVID-19 is caused by severe acute respiratory syndrome coronavirus type 2 (SARS-CoV-2), which is highly contagious and has had a serious impact on the lives of people around the world. The primary mode of transmission of SARS-CoV-2 is thought to be through respiratory droplets or contaminants. The common symptoms of initial infection are cough and fever, while complications, including acute respiratory distress syndrome and multi-organ failure, may occur after the disease has progressed, with a higher risk of complications in patients with cancer, respiratory disease, cardiovascular disease, and immunodeficiency-like diseases.^[1,2]^

Behcet’s disease (BD) is a nonspecific vascular inflammatory lesion with recurrent, self-limiting lesions. BD is closely related to oral ulcers, and about 90% of patients will have symptoms of recurrent oral ulcers, in addition, ulcers of varying degrees can appear in the patient’s eyes and genital skin. Also, as the disease progresses, the patient’s nervous system, blood vessels, and multiple organs can be involved.^[3,4]^ The cause of BD is still unclear, and some studies suggest that viral infection or allergic reaction to infectious factors is a factor that cannot be ignored. It has also been suggested that it is associated with a low-functioning fibrinolytic system, which leads to a slowing of blood flow, aggregation of red blood cells, and thrombosis, resulting in localized lesions due to tissue necrosis.^[5]^ However, the immune theory of pathogenesis is now generally accepted by researchers, and some studies have shown that the serum levels of immunoglobulins and immune complexes are elevated in patients with BD, and a variety of antibodies are produced, such as oral mucosal antibodies and anti-arterial wall antibodies, so it is speculated that the disease may be an autoimmune disease induced by some compound factors.^[6,7]^

The immunological theory of BD has been increasingly studied and more mechanisms of action at the cellular and molecular levels are being revealed. Deng et al found that^[6]^ human leukocyte antigen (HLA) genes are closely associated with susceptibility to autoimmune diseases, of which the HLA allele HLA-B51 is considered a genetic risk factor for BD, while other alleles such as HLA-A02 and HLA-A24 have been reported successively. Shimizu et al^[4]^ found that a large number of neutrophil infiltrates occur in the vicinity of early lesions in BD and that HLA-B51, as well as Th17 cells, are key factors in neutrophil activation, while peripheral blood of BD patients with Th1 and Th17 cells were significantly upregulated and the function of Th2 cells was suppressed. A study on BD with pulmonary lesions found an increased ratio of RORC/FOXP3 in bronchoalveolar lavage fluid of patients with BD, while the percentage of natural killer cells was significantly lower than in controls, and this phenomenon may be related to abnormal expression of perforin and granzyme in natural killer cells.^[8]^

The potential relationship between COVID-19 and autoimmune diseases has been explored as COVID-19 has been studied more and more. Liu et al^[9]^ found that many COVID-19 patients with significant clinical symptoms had detectable antibodies produced in autoimmune diseases, such as antinuclear antibodies, anti-neutrophil cytoplasmic antibodies, and antiphospholipid antibodies. However, patients with positive autoantibodies to COVID-19 often have a poor prognosis. Although there are relatively few case studies on COVID-19 with systemic lupus erythematosus (SLE), we found some clues about a potential association between the 2 in the study by Najafi et al^[10]^ Elevated levels of IL-17, IL-23, IFN-α, and γ in patients with SLE are often the cause of severe clinical symptoms in patients, and these factors also influence the prognostic status of patients with COVID-19. Some studies in patients with more severe COVID-19 have shown cross-reactivity with autoantigens and suggest that the virus shares an immunoreactive epitope with HLA-B series antigens.^[11,12]^ In many studies on BD, we have also found that the HLA-B family of antigens tends to be significantly elevated in BD patients,^[13]^ a finding that reinforces our concern that BD patients are more susceptible to COVID-19 infection than the general population, and even further exacerbates the worsening of clinical symptoms in BD.

COVID-19 can trigger a systemic inflammatory response when the expression levels of peripheral blood mononuclear cell (PBMCs) are also elevated and influence the expression of inflammatory factors that are associated with a variety of diseases related to the immune system. Inflammatory factors such as IL-2R, IL-6, and TNF-α have been reported to be significantly increased in PBMCs in COVID-19 patients with severe disease, while IL-10 exhibits fluctuations with disease progression, and IL-10 usually also inhibits the synthesis of cytokines such as IL-2, IL-6, and IL-8, thereby suppressing the immune response, in counteracting cytokine storm-mediated severe disease plays a key role.^[14]^ One of the main manifestations of BD is an increase in PBMCs in the body. Its main pathological mechanism is due to a dysregulation of the body’s immune system, which can generate an immune response by recognizing healthy cells or antigens, producing antibodies, and inducing a large number of monocytes around damaged cells or antigens. This increase in PBMCs can lead to dysfunction of the blood system, which can cause a variety of symptoms.^[15]^ Some studies have shown that major cellular inflammatory factors, play an important role in the pathogenesis of BD by stimulating the proliferation of inflammatory cells, which subsequently allows the release of more inflammatory factors, leading to apoptosis of tissue cells and triggering tissue damage.^[16,17]^ Although little is known about the mechanisms associated between BD and COVID-19, COVID-19 is often accompanied by changes in factors associated with autoimmune diseases.

Through this study, we can find more clues between the development of COVID-19 and BD, and provide more targeted treatment ideas for COVID-19 patients with autoimmune disease features. But the development of the disease is a long-term and dynamic process, and the interconnections arising between the two will involve more genes and biological response processes. Therefore, we will study PBMCs samples from patients with BD and COVID-19 by bioinformatics techniques and systems biology methods to further uncover the potential relationship between the two.

## 2. Materials and Methods

### 2.1. Data search and collation

To determine the relationship between COVID-19 and BD, we searched the GEO database (https://www.ncbi.nlm.nih.gov/geo/) for microarray and RNA-seq datasets of relevant studies.^[18]^ For the COVID-19 dataset, we chose GSE152418, which contains 17 samples of PBMCs from COVID-19 patients with clinical symptoms and 17 normal-group PBMCs samples. This dataset was transcriptional profiling by high-throughput sequencing method on Illumina NovaSeq 6000 platform for RNA sequence extraction. The dataset for BD is GSE198533, which contains 9 samples of PBMCs from BD patients and 10 samples of PBMCs from the normal group. The dataset was transcriptional profiling by high-throughput sequencing methods on Affymetrix Human Genome U133A Array and Illumina NovaSeq 6000 platforms.

### 2.2. Expression and screening of common differential genes between COVID-19 and BD

We used R to obtain differentially expressed genes in samples of PBMCs from COVID-19 patients as well as BD patients, controlled the false discovery rate using the limma package with Benjamini–Hochberg correction as well as the IMPUTE package, and applied screening criteria (*P* < .05) to detect the expression of relevant genes from the dataset. And the common differential genes were obtained by the VennR package.

### 2.3. PPI network analysis and sub-network analysis

Protein-protein interaction (PPI) networks consist of interactions between proteins, which play an important role in important life processes such as signaling, regulation of gene expression, metabolism of energy and substances, and cell cycle regulation in organisms.^[19,20]^ We used the String online database (https://cn.string-db.org/) to construct the protein interaction network of common differential genes to characterize the biofunctional interaction between COVID-19 and BD, after which the network data from the String database were downloaded and imported into Cytoscape for further PPI network experimental study.

The Mcode plugin in Cytoscape is a network analysis tool that identifies the most closely connected nodes based on the correlation of their interconnections. The results of this analysis are presented as clusters or groups of nodes, and the analysis parameters can be adjusted to obtain more detailed clusters of clusters.^[21]^ In this study, we imported network data and used the Mcode tool to classify nodes into different clusters to further present the interactions between more closely connected nodes in the network.

### 2.4. GO and pathway enrichment analysis

Gene enrichment analysis is one of the most important analytical works in bioinformatics techniques for classifying common biological response phenomena, usually with a focus on pathway enrichment studies and functional enrichment studies.^[22]^ In this study, we choose biological processes (BP), cellular components (CC), and molecular functions (MF) for functional enrichment analysis. For the pathway enrichment analysis, we integrated information related to the Kyoto Encyclopedia of Genes and Genomes database to obtain the common signaling pathway between COVID-19 and BD.

First, we used the “org.Hs.e.g..db” package to transform the ID numbers of duplicate differentially expressed genes, and the “clusterProfiler” package, the “enrichplot” package to perform gene ontology (GO) and pathway analysis. We used *P* < .05 as the screening index, and the entries were considered significantly enriched when the *P* value corresponding to GO and signaling pathway entries was less than .05.

### 2.5. Access to hub genes and functional analysis

PPI networks are usually composed of protein nodes as well as edges, where the nodes that are more closely related to other nodes are considered hub genes. Cytohubba, a plug-in for Cytoscape, can be used to score and rank PPI networks based on network characteristics. Cytohubba has several methods to study PPI networks from different perspectives, such as degree values.^[23]^ The degree value can reflect the connection count between each node in the network, and by calculating the degree value, we can gain insight into the network structure and relationships. Therefore, we use Cytohubba’s method to calculate the degree value to score and rank all the common differential genes and filter the top 15 hub genes in the PPI network.

GeneMANIA is a tool for gene research and analysis that can create visual networks that combine different types of genes, interacting functions, and databases, giving researchers insight into the molecular functions and interactions between different genes.^[24]^ In this study, we used the GeneMANIA online tool (http://www.genemania.org/) to build out co-expression networks of hub genes.

### 2.6. GO and pathway enrichment analysis of hub genes

We imported the list of hub genes into R and used the “org.Hs.e.g..db” package to convert the ID numbers of duplicate hub genes, and the “clusterProfiler” package, the “enrichplot” package to perform GO and pathway analysis, and we used *P* < .05 as the screening index, when the *P* value of GO and signaling pathway entries was less than .05, these entries were considered to be significantly enriched.

### 2.7. Validation of hub genes expression in the COVID-19 and BD dataset

To further explore the potential relationship between COVID-19 and BD, we used R to analyze the differences in expression levels of hub genes. We utilized the BD dataset (GSE198533) and the COVID-19 validation dataset (GSE164805) for gene expression validation. GSE164805 contains 5 samples of PBMCs from the normal group as well as 10 samples of PBMCs from BD patients. The *t* test was used to compare the 2 data sets and was considered to be significantly different when the *P* value was less than .05.

### 2.8. Interactions between hub genes and TFs and miRNAs

Transcription factor (TF) is the protein that attaches to a specific gene and controls the rate of transcription of genetic information and is a key factor in the regulation of gene expression. We searched for the relationship between hub genes and related TFs through the Trrust database (https://www.grnpedia.org/trrust). The Trrust database is a database that records the regulatory relationships of TFs, presenting the relationships between TFs and corresponding target genes.^[25]^ In addition, to further explore the interaction relationship between hub genes and miRNAs, we extracted miRNAs associated with hub genes using miRTarbase database (https://miRTarBase.cuhk.edu.cn/),^[26]^ and presented the interaction network between them through network analysis, so as to further explore the potential relationship between TFs-hub genes-miRNAs and derive a valid biological hypothesis.

### 2.9. Interaction of hub genes-related TFs in COVID-19 and BD

To further understand the regulatory network of related TFs, we analyzed the interaction relationships between TFs using R. We utilized the BD dataset (GSE198533) and the COVID-19 dataset (GSE152418) for the validation of TFs interaction relationships.

### 2.10. Genes-diseases association analysis

The DisGeNET database is a genes-diseases association database, including the study of the molecular basis of specific human diseases and their complications, pathogenic gene characterization, assistance in the construction of hypotheses of drug therapeutic effects and adverse drug reactions, validation of disease candidate genes and evaluation of the performance of text mining methods.^[27]^ We further uncovered the relationships between hub genes and other diseases through the DisGeNET database (https://www.disgenet.org/), which can reveal potential interactions between COVID-19, BD, and common diseases.

### 2.11. Screening of hub genes-related drugs

In this study, the prediction of drug-target gene interactions is also a more important part. We screened the active drug components through the Drug Signature Database (DSigDB) in the Enrichr platform (https://maayanlab.cloud/Enrichr/), a comprehensive web-based data platform for gene enrichment analysis, which contains a large number of genomic libraries. The DSigDB database can be used to screen the molecular structures of targeted drugs associated with common differential genes, and accessing the DSigDB database through the Enrichr platform is a convenient and efficient way to do so.^[28,29]^ We used the above approach to uncover potential interactions between hub genes and active drug components between COVID-19 and BD.

## 3. Results

### 3.1. Expression and screening of common differential genes between COVID-19 and BD

**Figure 1.**
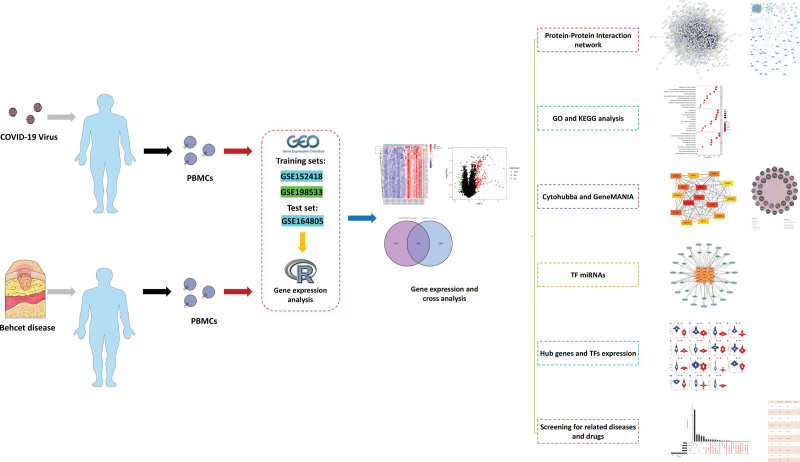
Research design flow chart. PBMC = peripheral blood mononuclear cell.

**Figure 2.**
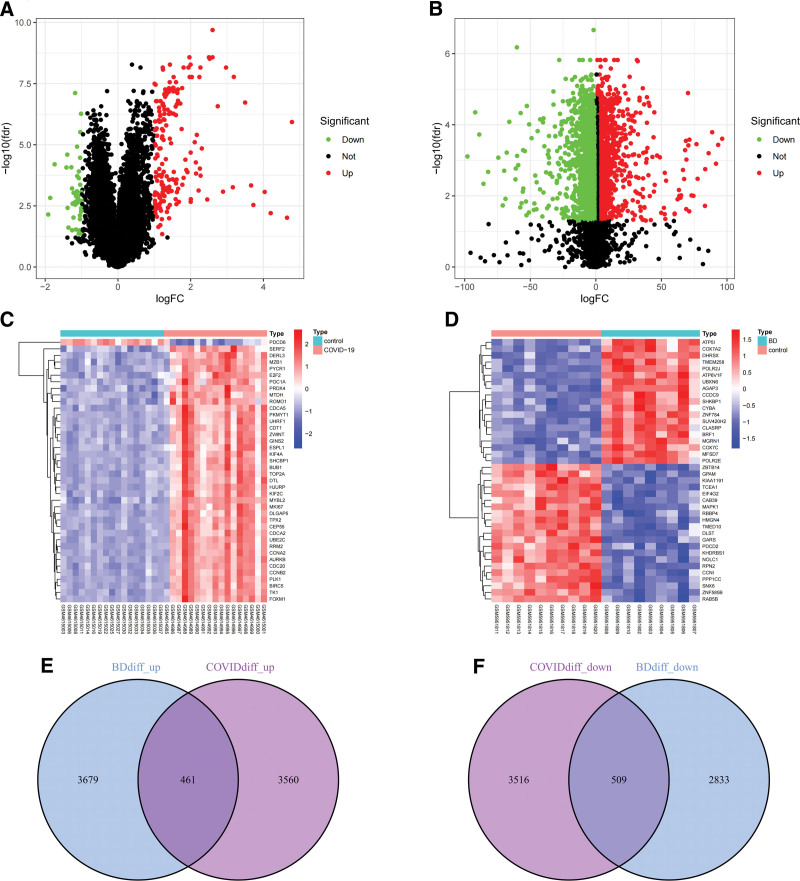
Heatmap, volcano diagram and Venn diagram. (A) The volcano diagram of GSE152418. (B) The volcano diagram of GSE198533. Up-regulated genes are marked in red, down-regulated genes are marked in green. (C) The heatmap of GSE152418. (D) The heatmap of GSE198533. Horizontal coordinates are sample names, vertical coordinates are differentially expressed genes. Up-regulated genes are marked in red, down-regulated genes are marked in blue. (E) The 2 datasets showed an overlap of 461 up-regulated differentially expressed genes. (F) The 2 datasets showed an overlap of 509 down-regulated differentially expressed genes.

The experimental flow diagram is shown in Figure 1. First, we used the human RNA-seq dataset and microarray dataset from the GEO database to analyze the expression of different genes related to PBMCs samples from COVID-19 patients and PBMCs samples from BD patients in turn. According to the screening criteria, we screened a total of 8046 differential genes from PBMCs samples of COVID-19 patients, including 4021 up-regulated genes and 4025 down-regulated genes. A total of 7482 differential genes of PBMCs samples from patients with BD were screened, including 4140 up-regulated genes and 3342 down-regulated genes. We mapped the volcanoes (Fig. 2A and B) and heatmap the most significantly differential genes for each dataset (Fig. 2C and D). Afterward, we obtained a total of 461 up-regulated differential genes and 509 down-regulated differential genes after cross-comparison analysis of differential genes by VennR package (Fig. 2E and F).

### 3.2. PPI network analysis and sub-network analysis

**Figure 3.**
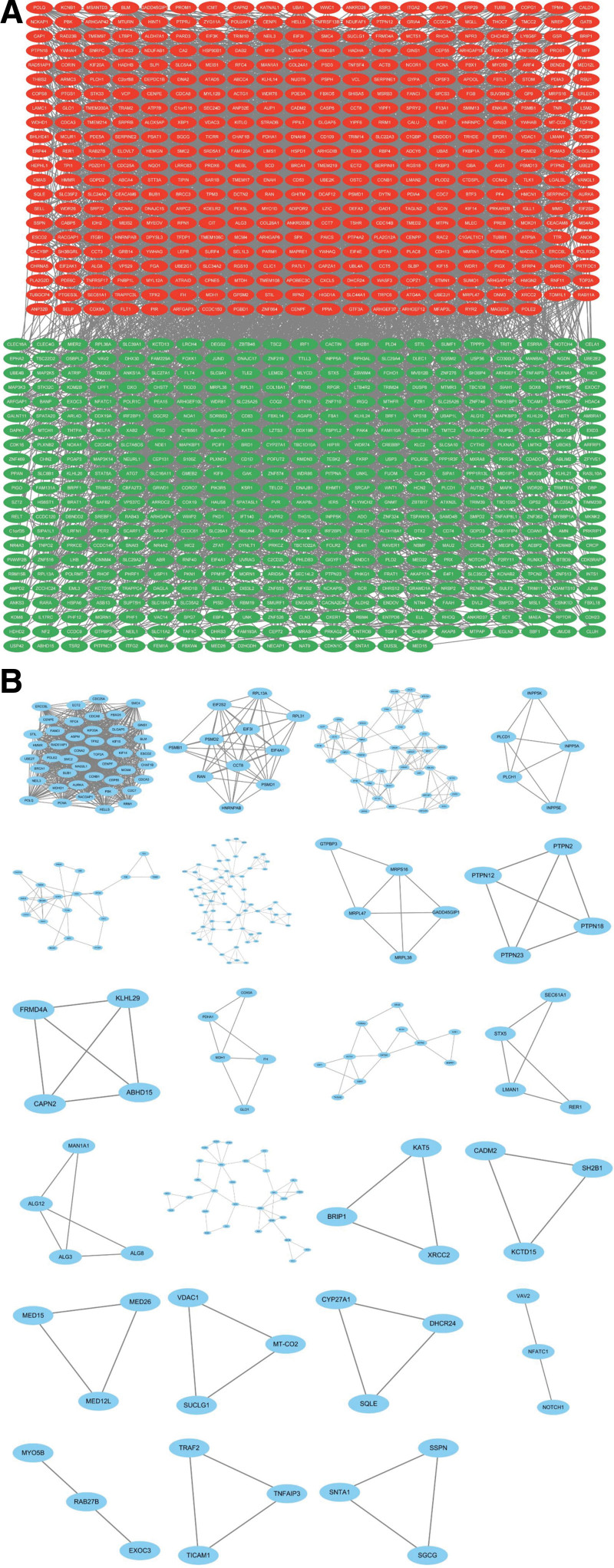
PPI network analysis. (A) Up-regulated and down-regulated genes are marked with different colors, Up-regulated genes are marked in red, down-regulated genes are marked in green. (B) Cluster analysis divided a total of 23 sub-networks. PPI = protein-protein interaction.

To further investigate the interactions between common differential genes, we imported the common differential genes into String online database to construct the PPI network, and after removing the genes without interactions, we obtained the PPI network with 894 nodes, 4940 edges, and a mean degree value of 11.1. After that, we imported the network data into Cytoscape and combined the data of differential gene expression levels to classify the common differential genes, where red indicates up-regulated genes and green indicates down-regulated genes (Fig. 3A). We used the Mcode analysis tool in Cytoscape to delineate a total of 23 gene sub-networks (Fig. 3B).

### 3.3. GO and pathway enrichment analysis

**Figure 4.**
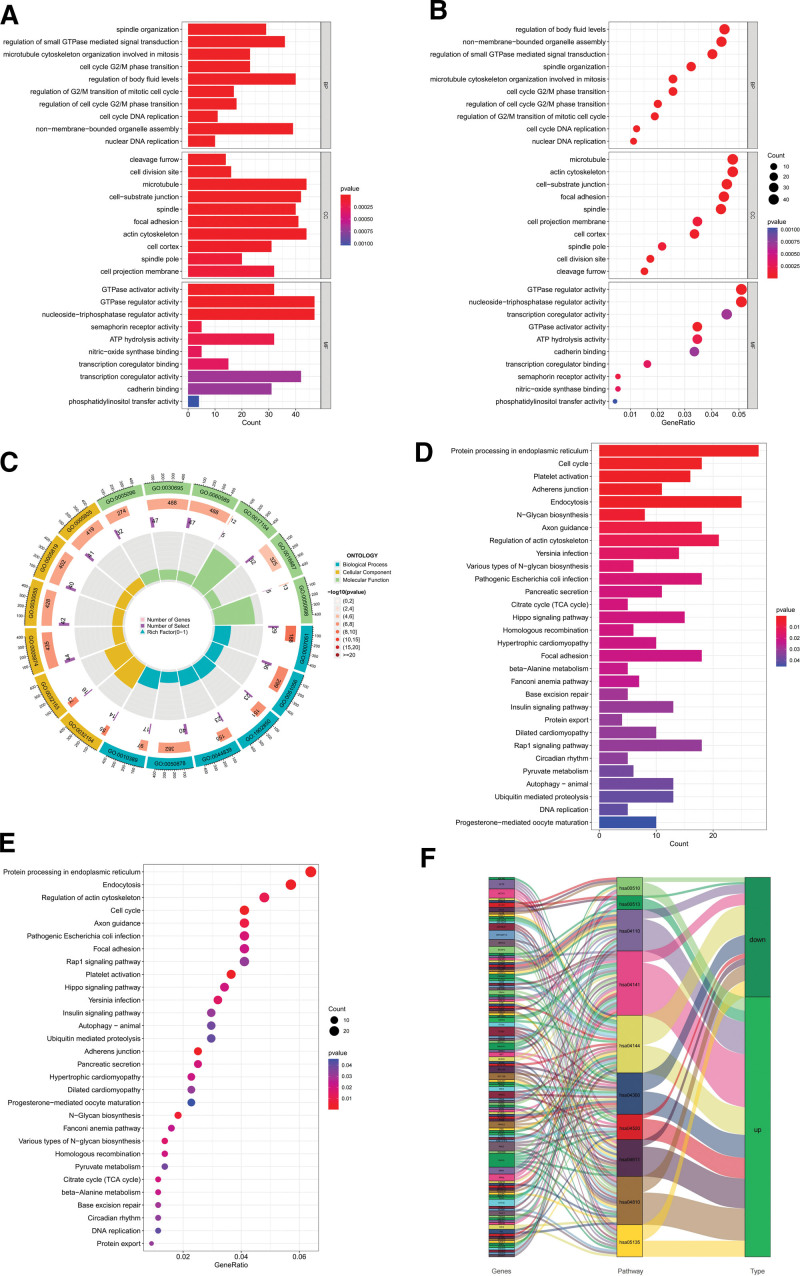
Gene ontology and pathway analysis of common differential genes. (A) Bar chart of Gene ontology analysis. (B) Bubble diagram of Gene ontology analysis. (C) Circle chart of Gene ontology analysis. (D) Bar chart of pathway analysis. (E) Bubble diagram of pathway analysis. (F) Sankey diagram of pathway analysis.

To further determine the biological significance of the common differential genes, and their association with signaling pathways, we selected 3 directions of BP, CC, and MF for GO analysis, and chose the GO database as the annotation source. Through the analysis, we obtained a total of 643 valid BP entries, 104 valid CC entries, and 102 valid MF entries. We summarized the top 10 entries for BP, CC, and MF, and plotted bar and bubble plots using R (Fig. 4A and B). To show the relationship of the differential genes corresponding to GO entries, we also plotted circle diagrams (Fig. 4C).

We integrated information from the Kyoto Encyclopedia of Genes and Genomes database to perform the analysis. We obtained 32 valid pathway entries through the pathway analysis, and we summarized the top 30 pathway entries and used R to drawbar and bubble diagrams (Fig. 4D and E). In addition, we used the R package to draw Sankey diagrams to show the relationship of genes corresponding to pathways (Fig. 4F).

### 3.4. Access to hub genes and functional analysis

**Figure 5.**
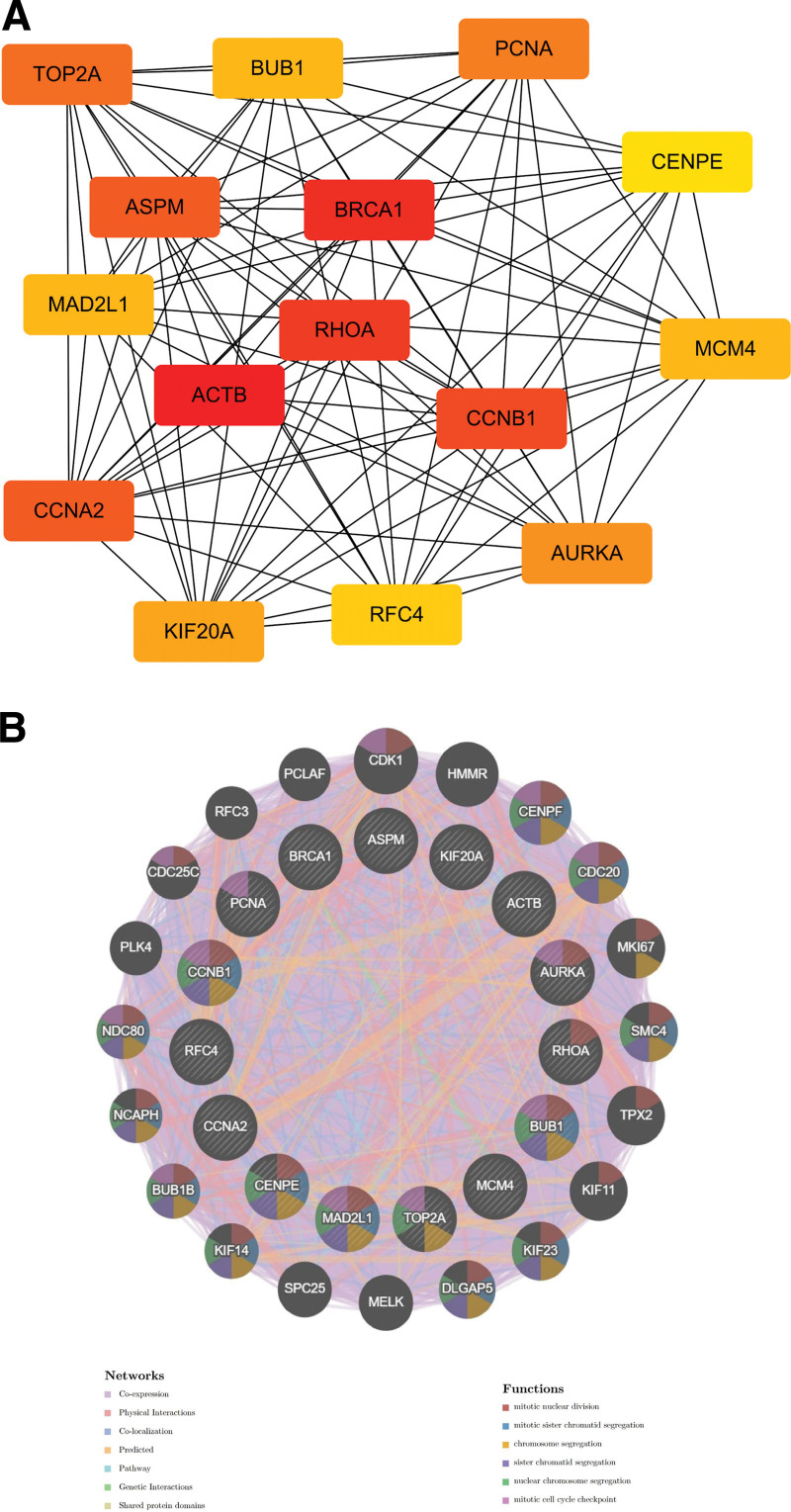
Screening of hub genes. (A) Hub genes screened by Cytohubba plugin in Cytoscape. (B) Hub genes and their co-expression genes were analyzed via GeneMANIA.

We combined Cytohubba plug-in in Cytoscape in PPI network analysis and classified the top 15 genes as hub genes by Degree algorithm, which are ACTB, BRCA1, RHOA, CCNB1, ASPM, CCNA2, TOP2A, PCNA, AURKA, KIF20A, MAD2L1, MCM4, BUB1, RFC4, and CENPE (Fig. 5A), and these hub genes may be potential biomarkers that can inform the study of novel therapeutic strategies for the disease.

Based on the GeneMANIA database, we analyzed the co-expression network of hub genes, which presented complex functions when genes interacted. Among them, Co-expression accounted for 84.98%, Physical Interactions for 6.92%, Co-localization for 2.97%, Predicted for 2.89%, Pathway for 1.12%, Genetic Interactions for 0.96%, and Shared protein domains accounted for 0.15% (Fig. 5B).

### 3.5. GO and pathway enrichment analysis of hub genes

**Figure 6.**
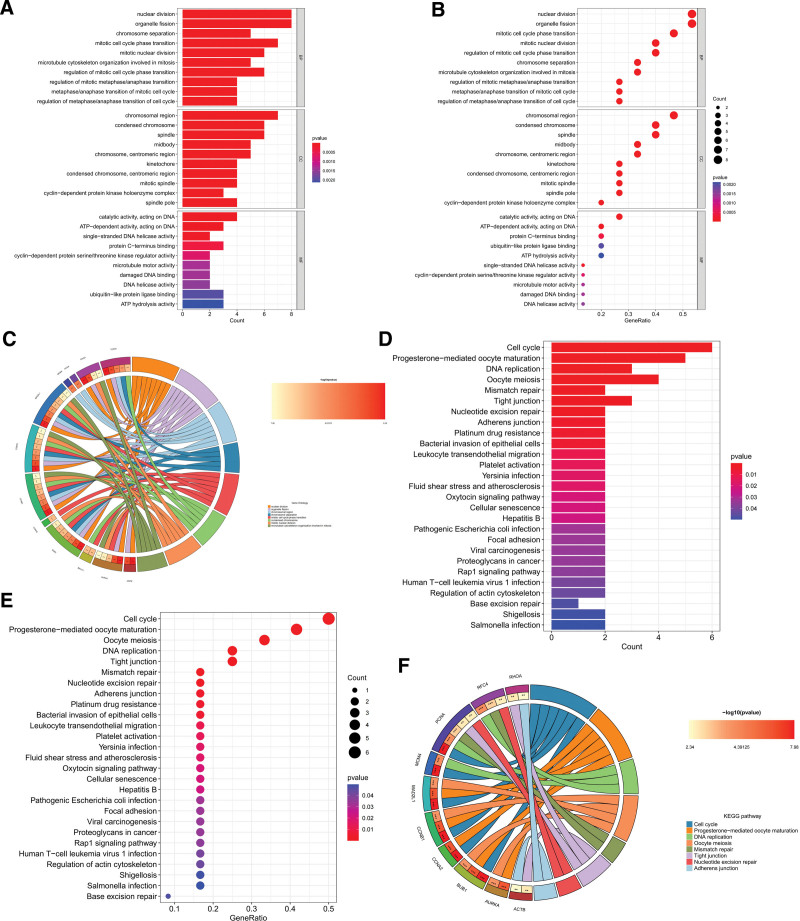
Gene ontology and pathway analysis of hub genes. (A) Bar chart of Gene ontology analysis. (B) Bubble diagram of Gene ontology analysis. (C) Circle chart of Gene ontology analysis. (D) Bar chart of pathway analysis. (E) Bubble diagram of pathway analysis. (F) Circle chart of pathway analysis.

To verify the biological significance of hub genes and the relationship of interactions, we still chose 3 directions of BP, CC, and MF for GO analysis, through which we obtained 464 valid BP entries, 58 valid CC entries, and 43 valid MF entries and plotted bar and bubble plots using R (Fig. 6A and B). To further present the relationship between pathways corresponding to target genes, we screened the top 8 entries with the strongest variability and plotted circle plots (Fig. 6C).

In addition, we also performed pathway analysis of hub genes and obtained a total of 27 valid pathway entries, and plotted bar and bubble diagrams (Fig. 6D and E). To verify the relationship between pathways corresponding to target genes, we filtered the top 8 pathway entries and plotted circle diagrams (Fig. 6F).

### 3.6. Validation of hub genes expression in the COVID-19 and BD dataset

**Figure 7.**
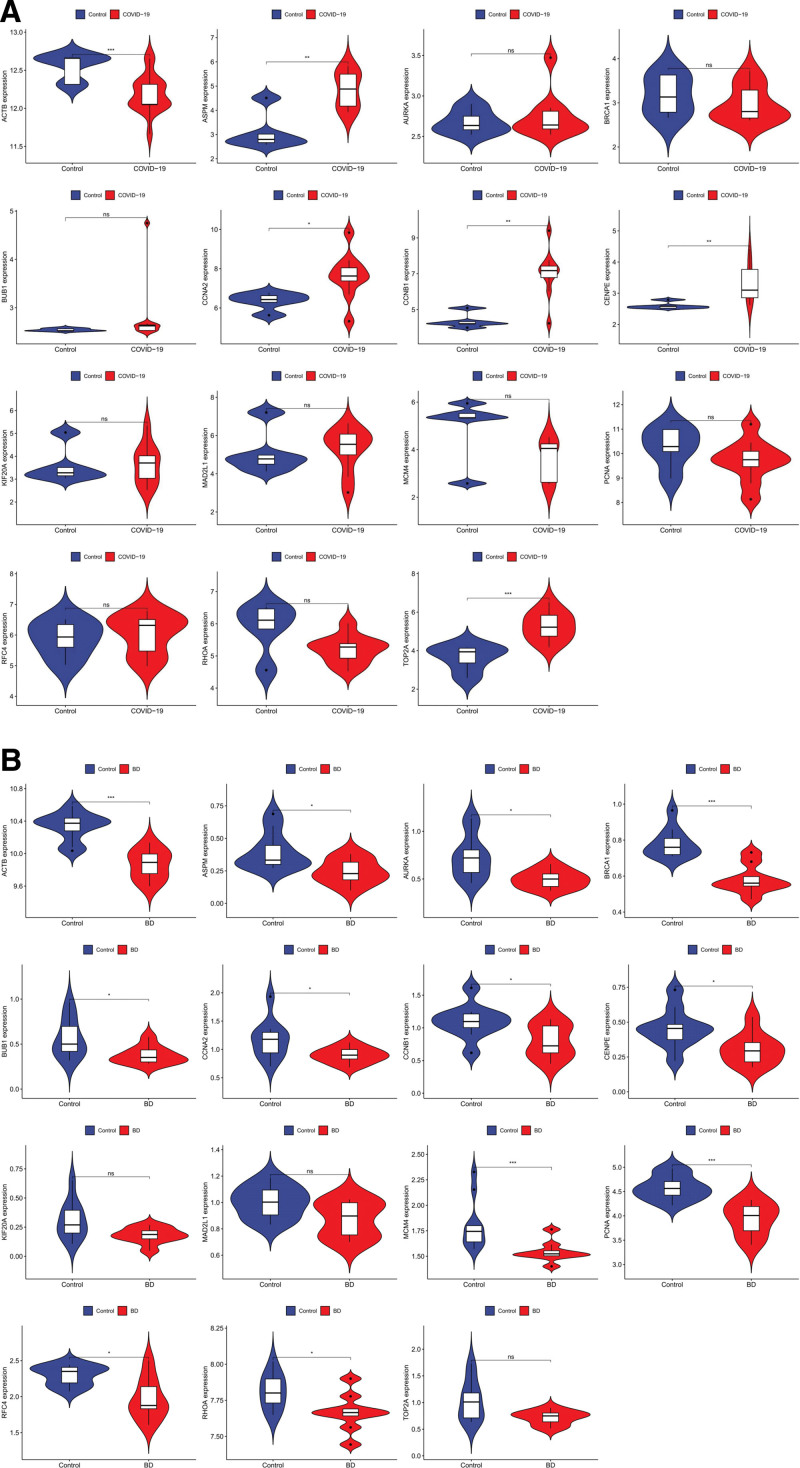
The expression level of hub genes. The comparison between the 2 sets of data uses the mean *t* test. *P* < .05 was considered statistically significant. *P < .05; ***P < .001. (A) The expression level of hub genes in GSE164805. (B) The expression level of hub genes in GSE198533.

To verify the reliability of hub genes expression levels, we selected the COVID-19 validation dataset for analysis with the BD dataset. From the results, it can be seen that the expression levels of hub genes were elevated in the COVID-19 samples compared with the normal group, except for ACTB. Among which the statistically significant hub genes were ACTB, ASPM, CCNA2, CCNB1, CENPE, and TOP2A (Fig. 7A). In contrast, in the BD dataset, the expression levels of hub genes were generally decreased compared with the normal group, among which the statistically significant hub genes were ACTB, ASPM, AURKA, BRCA1, BUB1, CCNA2, CCNB1, CENPE, MCM4, PCNA, RFC4, and RHOA (Fig. 7B). We screened the common hub genes ACTB, ASPM, CCNA2, CCNB1, and CENPE for genes-diseases network analysis and drug screening.

### 3.7. Interactions between hub genes and TFs and miRNAs

**Figure 8.**
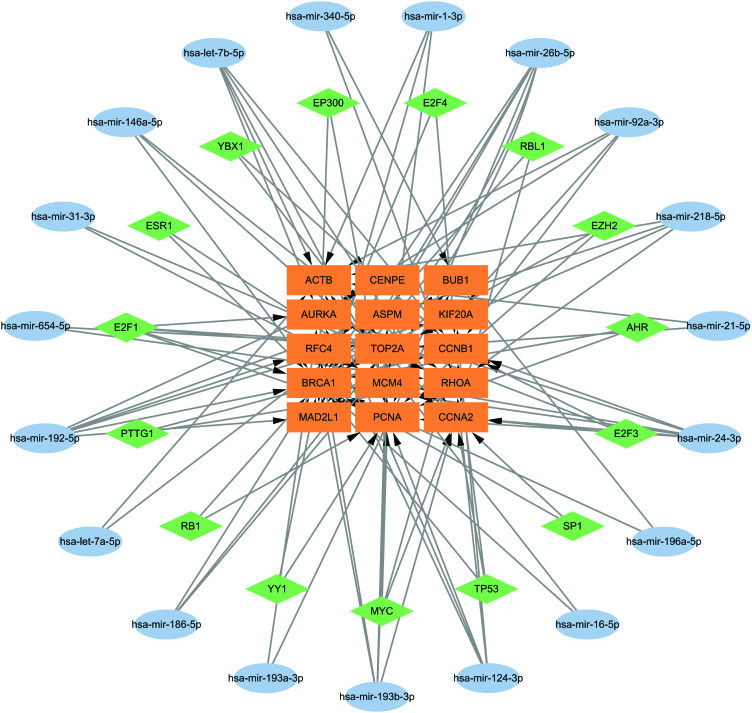
The regulatory network of hub genes and related miRNAs and TFs. TF = transcription factor.

To further identify changes at the transcriptional level and understand the relationship between TFs and hub genes, we performed network analysis between the 2 through the Trrust database, and the results showed that a total of 15 TFs were found to be associated with linked genes. Meanwhile, we analyzed the relationship between miRNAs and hub genes through the NetworkAnalyst platform, and we found 19 regulatory signals of miRNAs associated with hub genes (Fig. 8), which also indicates that the correlation between genes is a complex process regulated by different TFs, miRNA signals.

### 3.8. Interaction of hub genes-related TFs in COVID-19 and BD

**Figure 9.**
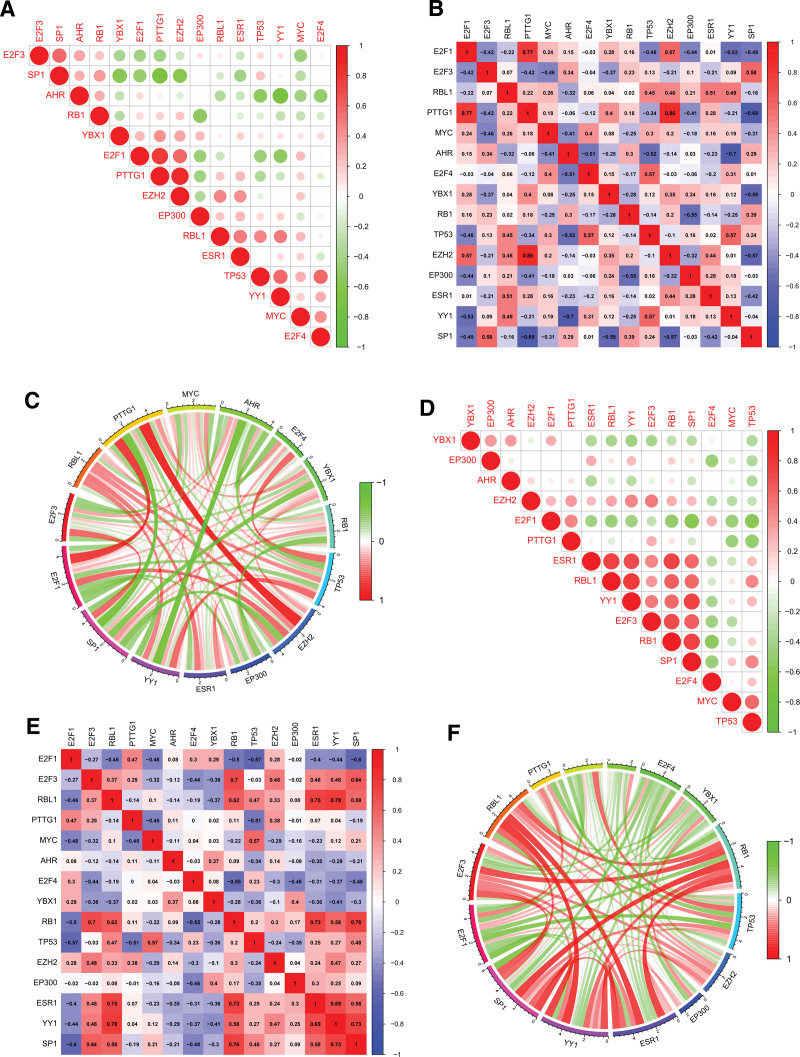
Validation of the interaction relationship between hub genes-related TFs. (A) Visualization of the correlation between TFs in GSE152418, red indicates positive correlation between TFs expression levels, green indicates negative correlation between TFs expression levels. (B) Quantification of correlation between TFs in GSE152418, red indicates positive correlation between TFs expression levels, blue indicates negative correlation between TFs expression levels, and the larger the value, the stronger the correlation. (C) The correlation between TFs in GSE152418 is shown by circle chart, red indicates positive correlation between TFs expression levels, green indicates negative correlation between TFs expression levels. (D) Visualization of the correlation between TFs in GSE198533. (E) Quantification of correlation between TFs in GSE198533. (F) The correlation between TFs in GSE198533 is shown by circle chart. TF = transcription factor.

To further characterize the regulatory role of TFs networks, we performed TFs interaction relationship analysis based on the COVID-19 and BD dataset. The results showed that the interaction relationship between EZH2 and PTTG1 was the strongest in the COVID-19 dataset, and the expression was positively correlated. The interaction between E2F1 and PTTG1 was also significant, and the expression of both was positively correlated. There was also a more significant interaction between YY1 and AHR, and the expression between them was negatively correlated (Fig. 9A–C). In the BD dataset, we found the strongest action relationship between YY1 and RBL1, and a positive correlation between them, while the action relationships between SP1 and RB1, ESR1 and RBL1 were also more significant and all showed a positive correlation (Fig. 9D–F).

### 3.9. Genes-diseases association analysis

**Figure 10.**
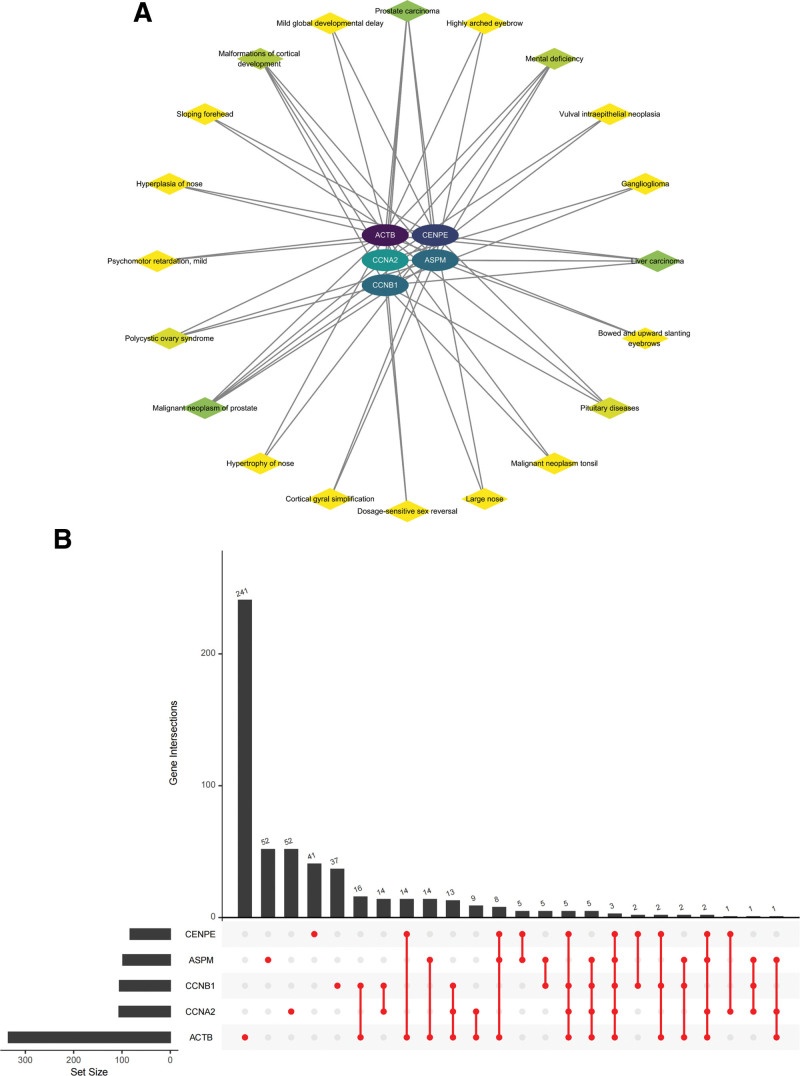
Genes-diseases association analysis. (A) Genes-diseases network, the darker the color, the stronger the association. (B) The upset chart shows the association of diseases with hub genes.

Different diseases are often associated with one or more common genes, and genes-diseases network analysis can lead to more therapeutic strategies for the treatment of related diseases. In the genes-diseases network analysis based on the DisGeNET database, we noticed that the association between hub genes was strong for cancer-like diseases (Prostate carcinoma, Liver carcinoma), Polycystic ovary syndrome, and Malformations of cortical development (Fig. 10A). We also plotted the upset chart between genes and diseases (Fig. 10B), and we found that the associated diseases were mainly concentrated in ACTB, where a total of 241 disease entries were enriched.

### 3.10. Screening of hub genes-related drugs

**Figure 11.**
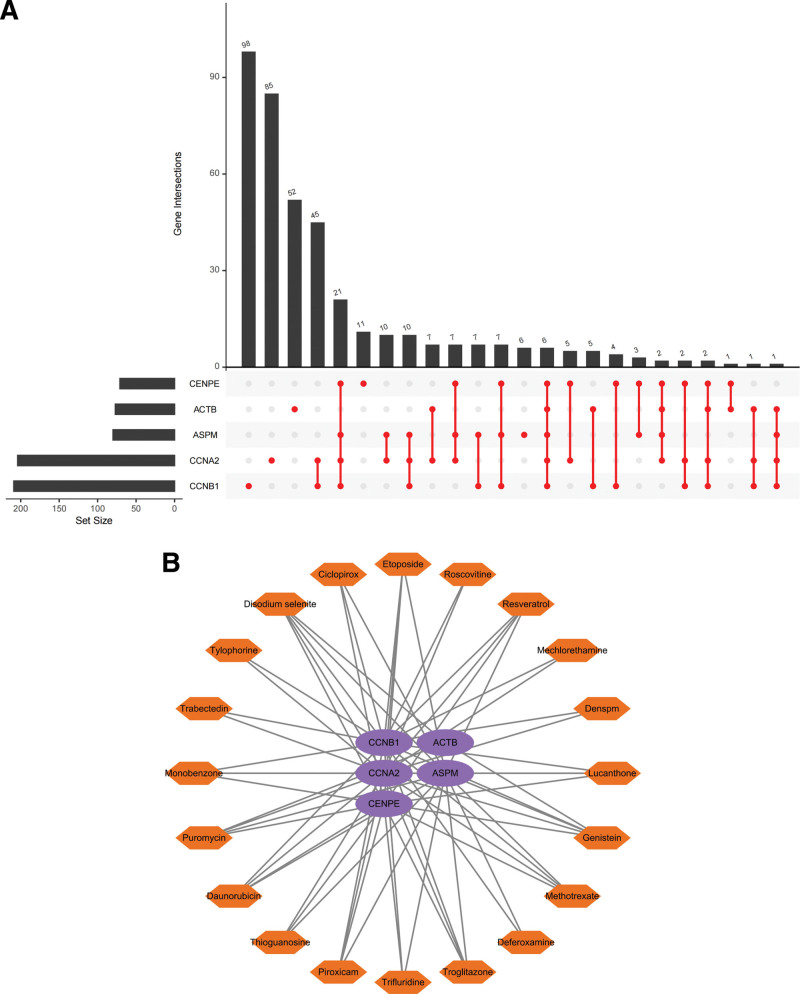
Genes-drugs association analysis. (A) The upset chart shows the association of drugs with hub genes. (B) Network relationships between top 20 drugs and genes.

Assessing the action relationship between drugs and target genes is essential to study receptor drug sensitivity. We entered ACTB, ASPM, CCNA2, CCNB1, and CENPE into the Enrichr platform and screened the interrelated drug molecules based on the DSigDB database, and a total of 398 effective drug molecules were screened and the genes-drugs upset chart was drawn (Fig. 11A), and we found that the drug molecules were mainly concentrated in CCNB1, with a total of 98 drug molecules were enriched in this area. We also listed the top 20 drug molecules with the strongest association (Fig. 11B), which can be considered as the drug molecules with the most significant effect on COVID-19 and PBMCs in BD patients, and we presented the details of the top 10 drug molecules screened from the DSigDB database in a table format (Table 1).

**Table 1:**
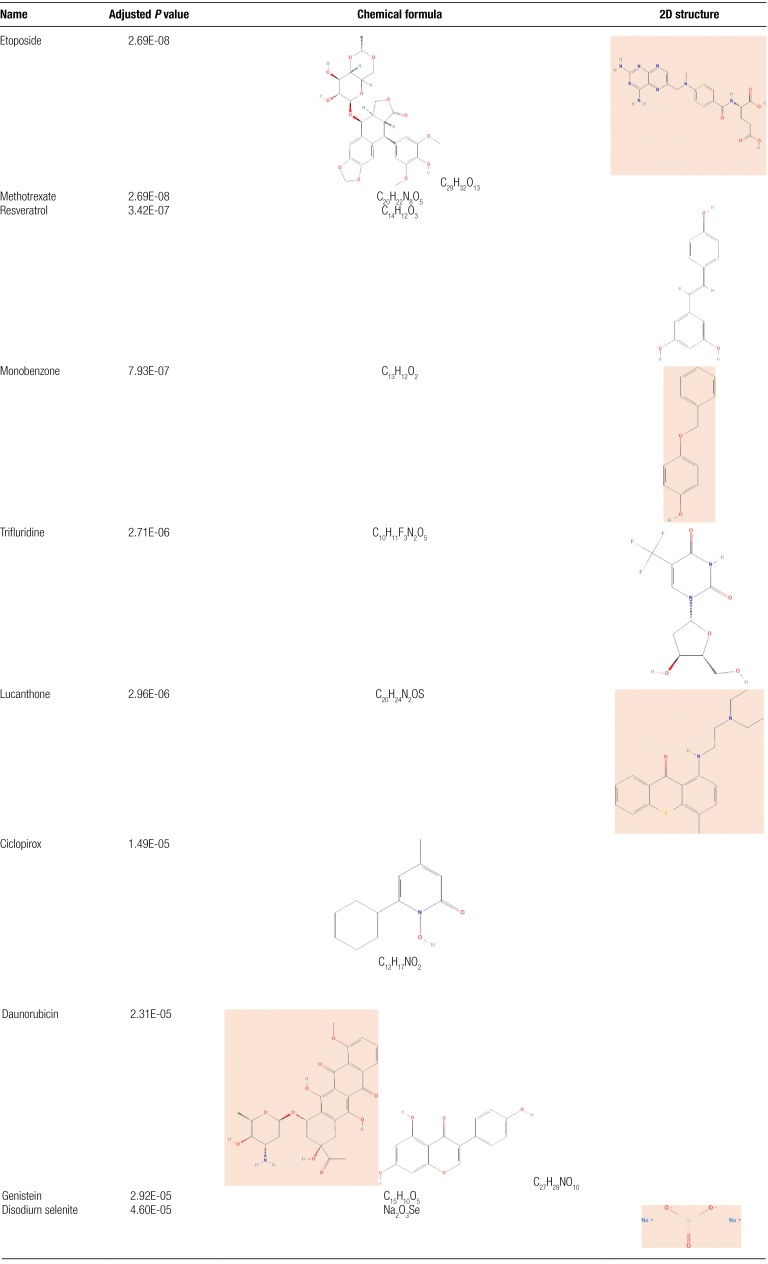
List of drug information.

## 4. Discussion

BD is a relatively rare chronic vasculitic disease that can lead to ulcers in the oral and genital areas, skin lesions, eye inflammation, and joint inflammation. Although it is now generally accepted that BD is an autoimmune disease, there are still studies that suggest that patients with BD have a weakened immune system and that this increases their susceptibility to COVID-19.^[30,31]^ However, the mechanism of action between BD and COVID-19 is still unclear, so in this study, we screened differentially expressed genes of the 2 diseases based on bioinformatics and discovered their interrelated signaling pathways to determine the interaction between BD and COVID-19.

We obtained the PPI network consisting of 894 genes by PPI analysis and compared the expression levels of hub genes in the BD dataset with the COVID-19 validation dataset, concluded that there were significant differences in the expression levels of ACTB, ASPM, CCNA2, CCNB1, and CENPE between the 2 diseases. Actin, cytoplasmic 1 (ACTB) is a highly conserved protein found in the cytoplasm and is a key component of the cytoskeleton that plays an important role in cell motility and cell division. In addition, it also plays an important role in the proper functioning of the immune system. It has been found that ACTB is aberrantly expressed in cancer and paracancerous tissues, ACTB expression is associated with immune cell infiltration and altered immune function. Some studies have shown that ACTB knockdown can inhibit the migration and invasion of head and neck squamous carcinoma cells through NF-κB and Wnt/β-catenin pathways.^[32]^ Chen et al^[33]^ found that such as ACTB, CXCR4, RHOA, and ITGAM genes can be used as hub genes in multiple sclerosis, and there is a significant downregulation of ACTB, RHOA, and ITGAM. Abnormal spindle-like microcephaly-associated protein (ASPM), the human homolog of the Drosophila “spindle anomaly” gene, has been found to encode multiple transcript variants of different subtypes of microcephaly. Although the exact relationship with COVID-19 has not been found in existing studies, a close association between this gene and lung cancer is known to exist. It has been found that^[34]^ ASPM is widely expressed in lung squamous cell carcinoma and co-expressed with CDK4, a cytokine-dependent kinase 4, which has been identified as a potential target against COVID-19, and it has been shown that inhibition of CDK4 activity prevents viral replication and allows systemic inflammatory symptoms to be alleviated. Moreover, CDK4 is essential for the progression of the cell cycle from G1 to S phase.^[35,36]^ The interaction between ASPM and CDK4 may play an important role in the replication and spread of the virus after the invasion of cells.

CCNA2 is the Cyclin-A2 gene, an A2 gene encoding a cell cycle protein on human chromosome 4. When a virus infects a cell, its genetic information can activate CCNA2, which promotes the cell cycle. In addition, overexpression of CCNA2 enhances cancer cell multiplication, as well as metastasis and invasion. It has been shown that CCNA2 can be a key target for the treatment of lung adenocarcinoma, and abnormal expression of CCNA2 plays a critical role in the development of lung adenocarcinoma.^[37]^ In addition, it has been shown that CCNA2 has a protective effect on innate immune responses and helps to reduce pathogen-induced inflammation and apoptosis, while CCNA2 is also critical for the regulation of immune-related gene expression.^[38]^ CCNB1, a member of the G2/mitotic-specific cyclin B1 family, controls the transition from G2 to M phase, is expressed in G2 phase and degraded in M phase. CCNB1 is also involved in transcriptional regulation in a cell cycle-dependent manner, and it plays an important role in the regulation of cell proliferation and apoptosis. It has been found that CCNB1 can act as a hub gene of COVID-19, and CCNB1 is closely related to signaling pathways such as P53, which is essential for DNA repair and apoptosis as a TF, and the interaction between CCNB1 and P53 also plays an important role in the regulation of the immune system.^[39,40]^ Mutations in CENPE, a motor protein that plays an important role in chromosome segregation during mitosis and meiosis, are commonly thought to be closely associated with chromosomal instability and some genetic diseases. However, some studies have shown that abnormal expression of CENPE can also exist in some autoimmune diseases. Kullmann et al^[41]^ found that in synovial fibroblasts of rheumatoid arthritis patients, the expression of CENPE showed a tendency to be reduced, and the motif of CENPE also showed significant homology with JUN and Fos gene products. In addition, it was found that^[42]^ CENPE was highly expressed in lung adenocarcinoma tissues, the proliferation of lung adenocarcinoma cells was inhibited by suppression of CENPE expression, and the expression of FOXM1 was also directly correlated with the expression of CENPE. It has also been found that FOXM1 was highly expressed in the lungs of COVID-19 patients, which also suggests that co-expression of FOXM1 with CENPE may have an important impact on the regulatory process of viral replication and spread.

We use GO and pathway analysis to further our understanding of the biological mechanisms underlying the interaction between BD and COVID-19. BP, CC, and MF describe the functions of the possible forms of genes, the cellular environment in which they are located, and in which biological processes they are involved. In this study, we found that the most strongly associated biological processes are nuclear division, organelle fission, and chromosome separation, which may be more active in the process of virus replication and spread. In autoimmune diseases, due to the abnormal function of the immune system, the phenomenon of nuclear division of immune cells often appears abnormal, and mainly manifests as organelle fission abnormalities, apoptosis abnormalities, and cell metabolism disorders, thus causing tissue cell damage and immune cell infiltration in the body. In addition, we screened the signaling pathways associated with hub genes, among which the most strongly associated pathways are cell cycle, progesterone-mediated oocyte maturation, and DNA replication, which also indicate a close connection between these key genes and the reverse transcription process of the virus and the division process of infected cells.^[43]^

We analyzed the relationship between common differential genes and TFs, and related miRNAs. TFs play a key role in gene regulation as well as RNA silencing, while miRNAs are also important in the development of diseases.^[44]^ In this study, we found that the TFs associated with key genes are E2F1, E2F3, RBL1, BRCA1, PTTG1, MYC, AHR, E2F4, YBX1, RB1, TP53, EZH2, EP300, ESR1, YY1, and SP1, and all of the above TFs have some connection with COVID-19 and BD. Through the TFs-genes-miRNAs network, we further clarified the molecular mechanism of the interaction between the 3.

We identified some miRNAs with a strong association with common differential genes, such as hsa-mir-193b-3p, hsa-mir-192-5p, hsa-mir-24-3p, hsa-mir-124-3p, and hsa-let-7b-5p. It has been reported that^[45]^ hsa-mir-193b-3p is closely related to lung adenocarcinoma, and its expression level is significantly elevated in lung adenocarcinoma tissues, and associated with signaling pathways such as PI3K-Akt signaling pathway, MAPK signaling pathway, and mTOR signaling pathway. The relationship between pneumonia and hsa-mir-192-5p has also been reported. One study showed that^[46]^ the expression level of hsa-mir-192-5p was significantly reduced in the blood of COVID-19 patients and differentially expressed in mild and severe patients. In addition, we found an association between hsa-mir-24-3p and pleural metastatic lung cancer, and some studies have shown that^[47]^ hsa-mir-24-3p may contribute to the metastasis and occurrence of lung cancer. We also found the basis for the association of hsa-mir-124-3p and hsa-let-7b-5p with the developmental process of COVID-19.^[48,49]^

We performed genes-diseases analysis to predict the association between hub genes and related diseases, and we found 284 diseases with significant association with hub genes, and we screened the top 20 diseases as the most significantly associated with hub genes. We constructed genes-diseases networks and found that such as liver carcinoma of cortical development was significantly associated with hub genes. Regarding the relationship between COVID-19 and neoplastic diseases, the research has also intensified in the last 2 years. it has been found that the immune response of COVID-19 patients is coordinated by pro-inflammatory factors such as IL-1, IL-6, and IL-8, which are also considered promoters of tumorigenesis.^[50]^ COVID-19 is also associated with the depletion of T cells, and the activation of oncogenic pathways, such as the JAK-STAT pathway, NF-κB pathway.^[51,52]^ It has been shown that there is a potential relationship between liver carcinoma and COVID-19, and this relationship may be related to the aberrant expression of hub genes Hmox1, TLR4, ALB, TTR, and RBP4.^[53]^ In previous studies, Hmox1 has also been suggested as a novel target for the treatment of autoimmune diseases and it plays an important role in oxidative stress response.^[54]^

In the study of pharmacological treatment of COVID-19, we know that chloroquine and Ridgavir have a good effect on the prevention of COVID-19, while hydroxychloroquine and azithromycin can also stop the replication of the virus.^[55,56]^ Through this study, we identified a cell cycle-specific antitumor drug, Etoposide, which mainly acts on DNA topoisomerase II and impedes DNA repair. Etoposide is now known to be more effective in treating patients with severe COVID-19, and its mechanism of action may be related to inhibiting the activity of activated T cells and monocytes and reducing the production of inflammatory factors.^[57]^ The other drug with a stronger association, namely Methotrexate, which we currently known to be an immunosuppressive drug with suppressive effects on both humoral and cellular immunity and strong anti-inflammatory effects, is mainly used in rheumatoid arthritis and systemic lupus erythematosus.^[58,59]^ In addition, a study showed that when mice were given Methotrexate, the expression level of ACE2 in the lung and intestinal epithelium, which is the main target of the SARS-CoV-2 virus, was significantly reduced, suggesting that Methotrexate can reduce the chance of being infected by SARS-CoV-2 by decreasing the expression level of ACE2.^[60]^ Besides, we screened a polyphenolic compound, Resveratrol, which has been shown to regulate platelet activation and aggregation as well as coagulation cascade reaction, so it is expected to improve vascular thrombosis and systemic inflammation produced in COVID-19 severe disease.^[61]^ In summary, through gene-drug association analysis, we uncovered more targeted drug components, which also provide more therapeutic strategies for the subsequent treatment of related diseases.

## 5. Conclusion

We analyzed the potential interaction relationship between COVID-19 and BD by summarizing the transcriptome data, screened the differentially expressed genes. We screened out common differential genes, and extracted hub genes from PPI network data, and combined with GO and pathway analysis, we confirmed the potentially reciprocal relationship between COVID-19 and BD.

Through this study, we found that the relationship between the effects of BD and COVID-19 is complex and deserves further study. However, patients with BD are at an increased risk of developing severe COVID-19, and the effectiveness of conventional drugs for BD may be reduced, making the treatment of patients with both diseases more difficult. This study clarifies the interaction between BD and COVID-19 and provides some reference for clinical treatment options for patients with both COVID-19 and BD, and also provides more research ideas to find the interaction between COVID-19 and other autoimmune diseases.

## Author contributions

**Data curation:** Zhibai Zhao, Chenyu Zhou

**Formal analysis:** Zhibai Zhao, Chenyu Zhou, Yuan Fan

**Funding acquisition:** Yuan Fan

**Investigation:** Mengna Zhang, Ling Qian, Wenhui Xia

**Methodology:** Yuan Fan

**Project administration:** Yuan Fan.

**Resources:** Yuan Fan.

**Software:** Zhibai Zhao

**Supervision:** Yuan Fan.

**Validation:** Mengna Zhang, Ling Qian, Wenhui Xia.

**Visualization:** Ling Qian, Wenhui Xia.

**Writing – original draft:** Zhibai Zhao.

**Writing – review & editing:** Chenyu Zhou, Mengna Zhang, Ling Qian, Wenhui Xia.
